# Effect of lifestyle intervention plus rosiglitazone or placebo therapy on left ventricular mass assessed with cardiovascular magnetic resonance in the metabolic syndrome

**DOI:** 10.1186/1532-429X-13-65

**Published:** 2011-10-28

**Authors:** Stijntje D Roes, Reza A Dehnavi, Jos JM Westenberg, Hildo J Lamb, Bart JA Mertens, Jouke T Tamsma, Albert de Roos

**Affiliations:** 1 Departments of Radiology, Endocrinology and Medical Statistics, Leiden University Medical Center, Albinusdreef 2, 2333 ZA Leiden, The Netherlands

## Abstract

**Background:**

To evaluate the effect of lifestyle intervention in conjunction with rosiglitazone or placebo therapy on left ventricular (LV) mass, using cardiovascular magnetic resonance (CMR) in the metabolic syndrome.

**Methods:**

The present study was a pre-specified substudy of a double-blind randomized controlled trial evaluating the effect of lifestyle intervention in conjunction with rosiglitazone or placebo therapy on carotid artery atherosclerosis in the metabolic syndrome. From this original study population, 10 subjects from the placebo group and 10 from the rosiglitazone group were randomly selected. At baseline and follow-up (52 weeks), clinical and laboratory measurements were assessed and a CMR-examination was performed to evaluate LV mass indexed for body surface area (LV mass-I). Subsequently, the effect of therapy (rosiglitazone vs. placebo) and clinical and laboratory variables on LV mass-I was evaluated.

**Results:**

In both groups, body mass index, waist circumference, systolic and diastolic blood pressure significantly decreased during follow-up. Interestingly, LV mass-I significantly decreased in the placebo group (48.9 ± 5.3 g/m^2 ^vs. 44.3 ± 5.6 g/m^2^, p < 0.001) indicating reverse remodeling, whereas LV mass-I remained unchanged in the rosiglitazone group (54.7 ± 9.9 g/m^2 ^vs. 53.7 ± 9.2 g/m^2^, p = 0.3). After correction for systolic and diastolic blood pressure and triglyceride, the kind of therapy (rosiglitazone vs. placebo) remained the only significant predictor of LV mass-I reduction.

**Conclusions:**

Lifestyle intervention resulted in a reduction of LV mass-I in the metabolic syndrome, indicating reverse remodeling. However, rosiglitazone therapy may have inhibited this positive reverse remodeling.

**Trial registration:**

Current Controlled Trials ISRCTN54951661.

## Background

The metabolic syndrome is a clustering of cardiovascular risk factors including abnormalities in glucose and lipid metabolism, abdominal obesity and hypertension [1], and is associated with increased risk of cardiovascular morbidity and mortality [2,3]. Although the exact pathophysiologic mechanism underlying the metabolic syndrome is still unclear, insulin resistance is believed to play a central role in the development of the metabolic syndrome [1,4]. Intensive lifestyle intervention by exercise and weight loss has beneficial effects on cardiovascular outcome in these patients. Also, pharmacological agents have been developed to reduce insulin resistance [5].

Peroxisome-proliferator-activated receptors (PPARs) regulate gene expression in response to ligand binding [5-7]. Thiazolidinediones (rosiglitazone, pioglitazone and troglitazone) represent a group of insulin sensitizing agents that can act as such ligands of the nuclear transcription factor PPAR-γ [5]. After ligand binding, PPARs undergo specific conformational changes that allow for the recruitment of coactivator proteins [8]. Ligands differ in their ability to interact with coactivators, which explains the various biologic responses observed [6,7]. Clinical studies have shown that thiazolidinediones e.g. lower blood glucose levels by enhancing hepatic and peripheral glucose uptake and increase free fatty acid uptake and storage in adipose tissue (thereby decreasing free fatty acid uptake in other tissues) [5,9].

Previous studies revealed that patients with the metabolic syndrome often present with increased left ventricular (LV) mass (and LV hypertrophy) [10,11]. The Framingham Heart Study evaluated LV mass using echocardiography in relation to long-term clinical outcome, and observed that an increase in LV mass is an independent predictor of clinical events, including death, attributable to cardiovascular disease [12].

The effect of thiazolidinediones on LV mass has been studied previously in experimental and clinical settings, but is however still unclear [13-17]. These clinical studies used 2-dimensional echocardiography to assess LV function and LV mass [15,17]. Cardiovascular magnetic resonance (CMR) however, is a highly reproducible technique allowing for more accurate measurement of LV mass, enabling reduction of sample size [18].

Accordingly, the purpose of the present study was to evaluate the effect of lifestyle intervention in conjunction with rosiglitazone or placebo therapy on LV mass using CMR in subjects with the metabolic syndrome.

## Methods

The present study was a pre-specified substudy of a double-blind randomized controlled trial evaluating the effect of lifestyle in conjunction with rosiglitazone or placebo therapy on carotid artery atherosclerosis in subjects with the metabolic syndrome (Rosiglitazone versUs placeBo on the prevENtion of progression of atheroSclerosis, RUBENS trial).

For this trial, 116 Caucasian male subjects with increased waist circumference (≥ 94 cm) and elevated CRP levels (≥ 1.8 mg/L), and two other metabolic syndrome criteria according to the International Diabetes Federation criteria [19] were selected. Exclusion criteria included type 2 diabetes (fasting blood glucose ≥ 7 mmol/l), manifest cardiovascular disease, use of statins, steroids or non-steroidal anti-inflammatory drugs at baseline, heart failure (New York Heart Association class I or higher), QTc time interval of 450 ms or longer on baseline electrocardiogram (ECG), primary dyslipidemias, presence of potential hepatic disease (i.e. subjects with alanine aminotransferase, total bilirubin, or alkaline phosphatase levels exceeding 2.5 times the upper limit of the normal laboratory values), alcohol abuse (> 30 units/week) and CMR contraindications. The study consisted of two periods: the screening phase and a double-blind study period with a scheduled duration of 52 weeks. After the screening phase, eligible subjects were randomly assigned in a 1:1 ratio (using computer generated codes) to receive either daily therapy with 8 mg of rosiglitazone or placebo. The treatment was titrated: during the first eight weeks, the participants were treated with one tablet daily (rosiglitazone 4 mg or placebo), which was doubled after 8 weeks. Subsequently, all subjects were submitted to intensive lifestyle changes including a 1500 kCal diet. In addition, the participants were motivated to increase their level of daily physical activity aiming at an extra energy-expenditure of 270 kCal per day (i.e. a normal-pace walk of 30 minutes, three times daily). All subjects were closely monitored during the study by the study physician and vascular nurse at 8, 22, 36 and 52 weeks after randomization/baseline. Hypertension was treated using a predefined protocol: first with salt restriction followed by step-up pharmacological therapy if needed, starting with hydrochlorothiazide 12.5 mg followed by ACE inhibition.

From this original study population, 10 subjects from the placebo group and 10 subjects from the Rosiglitazone group were randomly selected (sample size calculation by Bellenger et al. [18]: using CMR, 9 subjects are needed to detect a 10 g change in LV mass with a power of 90% and an α error of 0.05, corresponding with a 4.5 g change in LV mass-I [average body surface area 2.2]). These 20 subjects underwent a CMR examination at baseline to evaluate LV mass (primary endpoint), LV volumes and LV systolic function. Assessment of clinical measurements (body mass index, waist circumference, blood pressure) and laboratory measurements (triglycerides, high-density lipoprotein cholesterol [HDL cholesterol], total cholesterol, fasting blood glucose, glycated hemoglobin, insulin, high-sensitivity C-reactive protein [hs-CRP]) were performed just prior to CMR. Insulin resistance was calculated according to the homeostatic model assessment method (HOMA-IR) [20]. CMR examination, and clinical and laboratory measurements were repeated after one year. Approval by the local ethics committee and informed consent were obtained.

### CMR data acquisition

CMR was performed on a 1.5T Gyroscan ACS-NT/Intera MRI scanner (Philips Medical Systems, Best, The Netherlands) equipped with powertrack 6000 gradients and 5-element cardiac synergy coil.

For evaluation of LV mass, LV volumes and LV systolic function, the heart was imaged in short-axis view from apex to base, with 10-12 imaging levels (dependent on heart size) using an ECG-triggered balanced turbo field-echo sequence [21]. Typical parameters were a field of view of 400 × 400 mm^2^, matrix of 256 × 256 pixels, slice thickness of 10.00 mm, no slice gap, flip angle of 50°, time to echo of 1.82 ms, and time to repeat of 3.65 ms. Temporal resolution was 25 to 39 ms.

### CMR data analysis

Data were analyzed with MASS software developed at our institution (Medis, Leiden, the Netherlands). Analysis was performed by the same researcher (initials S.D.R.) with more than five years experience in CMR. To determine LV mass, LV systolic function and volumes, endocardial and epicardial borders were manually traced on the short-axis cine images. Papillary muscles were regarded as part of the ventricular cavity. LV mass, LV end-systolic volume (LV ESV), LV end-diastolic volume (LV EDV) and ejection fraction (EF) were assessed. LV mass, LV EDV and LV ESV were corrected for body surface area (yielding LV mass-I, LV EDV-I and LV ESV-I).

### Statistical analysis

Continuous variables were tested for normal distribution (by evaluation of normal Q-Q plots of residuals obtained after correction for between group effects) and expressed as mean ± standard deviation (SD) or median (interquartile range). Differences at baseline and at follow-up between groups (rosiglitazone vs. placebo group) were analyzed using the unpaired *t*-test, Mann-Whitney *U-*test (numerical data) or Fisher's exact test (categorical data) as appropriate. Variables at baseline were compared to follow-up using the paired *t*-test, Wilcoxon singed rank test (numerical data) or McNemar's test (categorical data). Changes in variables from baseline to follow-up between subjects who received rosiglitazone and placebo therapy were compared using the unpaired *t*-test or Mann-Whitney *U*-test. Non-normally distributed variables were log transformed for further analysis. Furthermore, the relation between the kind of therapy (rosiglitazone vs. placebo) and changes in LV mass-I (primary endpoint), and alterations in clinical and CMR variables between baseline and follow-up and changes in LV mass-I (primary endpoint) were evaluated. (Pearson's correlation coefficients (r), p-values reported). Subsequently, variables with a significant relation or trend towards a significant relation with LV mass-I (p < 0.10) were included in a multiple linear regression model to evaluate the relation between these variables and LV mass-I. Furthermore, in order to correct for possible differences in LV mass-I at baseline, we have included this variable in the multiple linear regression model.

## Results

### Clinical and laboratory characteristics

At baseline, no differences in clinical and laboratory variables were observed between subjects who were randomized for rosiglitazone or placebo therapy. Variables at baseline and follow-up for the subjects who received rosiglitazone therapy and subjects who received placebo therapy are presented in Table 1. For both groups, body mass index, waist circumference, systolic and diastolic blood pressure significantly decreased from baseline to follow-up. In the placebo group, no significant change was observed in laboratory variables, except for a decrease in insulin (from 11.5 (9.3) to 7.0 (5.8), p = 0.007) and HOMA-IR (from 1.6 (1.1) to 0.9 (0.8), p = 0.007). In the rosiglitazone group, the level of triglycerides, insulin, HOMA-IR and hs-CRP significantly decreased, whereas HDL cholesterol significantly increased.

**Table 1:** Clinical and laboratory variables at baseline and follow-up

Clinical variables	Rosiglitazone therapy			Placebo therapy			Rosiglitazone therapy	Placebo therapy	
	Baseline	Follow-up	P-value	Baseline	Follow-up	P-value	Δ baseline-follow-up	Δ baseline-follow-up	P-value
Age (yrs)	60 ± 6	61 ± 6	< 0.001	57 ± 5	58 ± 5	< 0.001			
Body mass index (kg/m^2^)	30.3 ± 4.5	28.7 ± 4.8	0.001	31.4 ± 4.9	29.2 ± 3.8	0.022	-1.6 ± 1.0	-2.2 ± 2.5	0.5
Waist circumference (cm)	112 ± 11	104 ± 14	0.001	111 ± 12	102 ± 10	0.005	-8 ± 5	- 8 ± 7	0.9
Systolic blood pressure (mmHg)	154 ± 23	136 ± 15	0.021	149 ± 14	135 ± 11	0.001	-18 ± 21	-14 ± 9	0.6
Diastolic blood pressure (mmHg)	91 ± 11	78 ± 8	0.001	89 ± 6	78 ± 5	< 0.001	-13 ± 9	-11 ± 5	0.6
Antihypertensive drugs, N (%)	2 (20)	4 (40)	0.5	4 (40)	4 (40)	1.0			
									
**Laboratory variables**									
Triglycerides (mmol/l)	1.8 ± 0.5	1.4 ± 0.3	0.008	1.9 ± 0.7	1.7 ± 0.6	0.3	-0.4 ± 0.4	-0.2 ± 0.5	0.3
HDL cholesterol (mmol/l)	1.2 ± 0.2	1.4 ± 0.2	0.002	1.2 ± 0.2	1.2 ± 0.1†	0.7	0.2 ± 0.2	0.02 ± 0.2	0.024
LDL cholesterol (mmol/l)	3.8 ± 0.8	4.3 ± 0.6	0.079	3.9 ± 0.4	3.8 ± 0.5	0.7	0.6 ± 0.9	-0.06 ± 0.4	0.063
Total cholesterol (mmol/l)	5.8 ± 0.9	6.3 ± 0.7	0.1	5.9 ± 0.6	5.8 ± 0.6	0.5	0.6 ± 1.0	-0.1 ± 0.6	0.075
Fasting blood glucose (mmol/l)	5.0 ± 0.6	4.8 ± 0.7	0.098	5.7 ± 1.0	5.3 ± 1.1	0.071	-0.3 ± 0.5	-0.4 ± 0.6	0.6
HbA1c (%)	5.2 ± 0.6	5.3 ± 0.4	0.3	5.2 ± 0.4	5.3 ± 0.3	1.0	0.1 ± 0.3	0.0 ± 0.4	0.5
Insulin (mmol/l)*	11.5 (11.3)	6.0 (4.3)	0.034	11.5 (9.3)	7.0 (5.8)	0.007	-4.2 ± 5.0	-7.6 ± 7.3	0.3
HOMA-IR (mmol/l × mU/l)*	1.5 (1.5)	0.6 (0.5)	0.021	1.6 (1.1)	0.9 (0.8)	0.007	-0.6 ± 0.6	-1.1 ± 1.0	0.2
hs-CRP (mmol/l)*	2.4 (3.1)	1.6 (1.7)	0.047	2.6 (0.9)	2.6 (2.6)	0.6	-1.7 ± 2.5	7.0 ± 21.2	0.2

Data are expressed as mean ± SD. * Variables were non-normally distributed and therefore expressed as median (interquartile range); Mann-Whitney *U *test or Wilcoxon singed rank test were used for comparison between groups and between baseline and follow-up, respectively.

† p < 0.05 between subjects who received Rosiglitazone or placebo therapy at follow-up.

HbA1c: glycated hemoglobin, HDL cholesterol: high*-*density lipoprotein cholesterol, HOMA-IR: homeostasis model assessment of insulin resistance (fasting blood glucose (mmol/l) × fasting insulin (mU/l)/22.5), hs-CRP: high-sensitivity C-reactive protein, LDL cholesterol: low*-*density lipoprotein cholesterol.

### CMR variables

Table 2 shows the CMR variables at baseline and follow-up for subjects who received rosiglitazone or placebo therapy in conjunction to lifestyle intervention, respectively. Except for LVEDV-I (91 ± 12 vs. 78 ± 6 ml/m^2^, p = 0.007, resp. rosiglitazone and placebo), CMR variables were similar between the two groups at baseline. LV mass-I at baseline was not significantly different between both groups (p = 0.13). In both groups, LV EDV en LV ESV and LVEF did not significantly change between baseline and follow-up. Interestingly, no significant change in LV mass-I was observed in the rosiglitazone group (54.7 ± 9.9 g/m^2 ^vs. 53.7 ± 9.2 g/m^2^, p = 0.3), whereas LV mass-I significantly decreased in the subjects who were treated with placebo therapy (48.9 ± 5.3 g/m^2 ^vs. 44.3 ± 5.6 g/m^2^, p < 0.001). Accordingly, mean LV mass-I decreased 1.0 ± 2.5 g/m^2 ^in the rosiglitazone group vs. 4.6 ± 1.9 g/m^2 ^in the placebo group (p = 0.002).

**Table 2:** CMR variables at baseline and follow-up

	Rosiglitazone therapy			Placebo therapy			Rosiglitazone therapy	Placebo therapy	
	Baseline	Follow-up	P-value	Baseline	Follow-up	P-value	Δ baseline-follow-up	Δ baseline-follow-up	P-value
**CMR variables**									
LV EDV-I (ml/m^2^)	91.1 ± 11.8	92.3 ± 5.1	0.7	78.7 ± 6.4 †	80.7 ± 10.7‡	0.5	1.2 ± 10.4	1.9 ± 9.0	0.9
LV ESV-I (ml/m^2^)	38.3 ± 8.9	36.8 ± 5.0	0.4	32.0 ± 4.5	32.5 ± 7.8	0.8	-1.5 ± 5.9	0.5 ± 6.2	0.5
LV mass-I (g/m^2^)	54.7 ± 9.9	53.7 ± 9.2	0.3	48.9 ± 5.3	44.3 ± 5.6‡	< 0.001	-1.0 ± 2.5	-4.6 ± 1.9	0.002
LVEF (%)	58.1 ± 6.7	60.1 ± 4.9	0.095	59.3 ± 4.4	60.0 ± 5.7	0.6	2.0 ± 3.3	0.7 ± 3.9	0.4

† p < 0.05 between subjects who received rosiglitazone or placebo therapy at baseline.

‡ p < 0.05 between subjects who received rosiglitazone or placebo therapy at follow-up.

LV EDV-I: left ventricular end-diastolic volume indexed for body surface area, LV ESV-I: left ventricular end-systolic volume indexed for body surface area: LV mass-I: left ventricular mass indexed for body surface area, left ventricular, LVEF: left ventricular ejection fraction.

None of the alterations in clinical and laboratory variables during follow-up correlated significantly with the change in LV mass-I. Only a trend towards a significant correlation was observed between change in triglyceride, systolic and diastolic blood pressure between baseline and follow-up and reduction in LV mass-I (r = -0.392, p = 0.087, r = -0.392, p = 0.087 and r = -0.384, p = 0.095, respectively).

The multiple linear regression models to assess the relative effect of rosiglitazone therapy, systolic and diastolic pressure and triglyceride on LV-mass-I are presented in Table 3. After correction for systolic and diastolic blood pressure and triglyceride, the kind of therapy (rosiglitazone vs. placebo) remained the only significant predictor of the extent of LV mass-I reduction. Also, after correction for LV mass-I at baseline, the kind of therapy remained the only significant predictor of LV mass-I reduction.

**Table 3:** Multivariable association clinical and laboratory variables and LV mass-I reduction

		Β-coefficient	P-value^a^	R^2^	P-value^b^
**Model 1**				0.421	0.002

	Rosiglitazone therapy	3.6 (1.5-5.7)	0.002		

**Model 2**				0.516	0.002

	Rosiglitazone therapy	3.2 (1.1-5.3)	0.006		
	Δ systolic blood pressure	-0.03 (-0.12-0.06)	0.5		
	Δ diastolic blood pressure	-0.07 (-0.27-0.12)	0.4		
	Δ triglyceride	-0.68 (-.2-1.8)	0.6		

**Model 3**				0.611	0.01

	Rosiglitazone therapy	3.7 (1.7-5.7)	0.003		
	Δ systolic blood pressure	-0.01 (-0.09-0.08)	0.9		
	Δ diastolic blood pressure	-0.11 (-0.29-0.07)	0.3		
	Δ triglyceride	-1.0 (-3.3-1.3)	0.4		
	LV mass-I at baseline	-0.1 (-0.2-0.3)	0.2		

^a ^Level of significance of the association between the separate components of the model and reduction in LV mass-I.

^b ^Level of significance of the model.

LV mass-I: left ventricular mass indexed for body surface area

## Discussion

The main findings in the current study are as follows: intensive lifestyle intervention including a diet and physical exercise in conjunction with either rosiglitazone or placebo therapy, improves clinical characteristics such as body mass index, waist circumference, diastolic and systolic blood pressure. Furthermore, lifestyle intervention in conjunction with rosiglitazone or placebo therapy results in a significant decrease in insulin and insulin resistance (HOMA-IR). Also, in subjects who were randomised for rosiglitazone therapy, a significant increase in HDL cholesterol and a decrease in triglyceride and hs-CRP was observed, whereas no change in these variables was observed in subjects who used placebo therapy. Interestingly, lifestyle intervention in conjunction with placebo therapy resulted in a reduction of LV mass-I, whereas in subjects who were randomised for rosiglitazone therapy, LV mass-I remained unchanged.

### Clinical variables

All subjects in our study were submitted to lifestyle intervention including a diet and exercise, which resulted in reduction of body mass index and waist circumference. This lifestyle intervention also significantly lowered systolic and diastolic blood pressure regardless of therapy (rosiglitazone or placebo). It is well known that weight reduction lowers blood pressure [22-24]. Systemic reviews of studies evaluating the effect of rosiglitazone have indeed not found any beneficial effects of rosiglitazone on blood pressure, which is in line with the results of the present study [5,25].

### Laboratory measurements

In both groups, thus regardless of treatment, insulin levels and insulin resistance significantly improved during follow-up. A major contributor to the development of insulin resistance (and thus higher levels of insulin) is the overabundance of free fatty acids produced in adipose tissue [1]. These free fatty acids lead to higher glucose and insulin levels through different pathways. For instance, free fatty acids stimulate production of glucose by the liver and high levels of free fatty acids lead to reduction of insulin sensitivity by inhibition of insulin-mediated glucose uptake in peripheral tissues [1]. Accordingly, weight loss (thus reduction of adipose tissue) will reduce insulin levels and insulin resistance. Results from large clinical trials indeed showed that lifestyle changes significantly reduced the incidence of diabetes [26,27]. Thiazolidinediones act as insulin sentitizers by multiple mechanisms and a previous study in non-diabetic insulin resistant individuals indeed demonstrated lower insulin and improvement of insulin resistance after rosiglitazone treatment [28]. In the current study, no additional effect of rosiglitazone on insulin and insulin resistance could be observed, stressing the highly beneficial effects of lifestyle intervention, overshadowing the possible effect of rosiglitazone on these parameters.

The finding that rosiglitazone increases HDL cholesterol is in accordance with previous clinical trials evaluating the effect of rosiglitazone on lipid profile in patients with type 2 diabetes, in which HDL cholesterol levels increased approximately 10 percent [29-31]. These studies also found an adverse effect on low-density lipoprotein (LDL) cholesterol levels after rosiglitazone therapy. Although no significant change could be observed in our study, LDL cholesterol levels indeed increased in patients using rosiglitazone therapy. In the group using rosiglitazone, a significant reduction in triglycerides was noted. Previous studies revealed controversial results on the effect of rosiglitazone on triglycerides; some studies showed a similar decrease in triglycerides as was observed in the current study, whereas others found no effect on triglyceride level [30,32,33].

### Left ventricular mass

Previous studies showed that subjects with the metabolic syndrome often present with increased LV mass (and LV hypertrophy) [10,11] The Framingham Heart Study evaluated LV mass using echocardiography and observed that increased LV mass is an independent predictor of clinical events such as heart failure, ischemia, ventricular arrhythmia, and sudden cardiac death [12]. Accordingly, reduction of LV mass is important for improvement of clinical outcome. Several studies reported that weight loss is associated with a reduction in LV mass [34-36]. A recent study by Rider et al. [35] evaluated obese patients who underwent bariatric surgery, and found a regression of LV hypertrophy in these patients. The results from the current study show LV mass-I significantly decreased in subjects who underwent extensive lifestyle intervention (resulting in weight reduction) in conjunction with placebo therapy. Accordingly, intensive lifestyle intervention including a diet and exercise results in reverse remodeling of the LV in subjects with the metabolic syndrome. However, the use of rosiglitazone may have inhibited this beneficial effect of lifestyle intervention, as LV mass-I remained unchanged in this group.

The effect of rosiglitazone therapy on LV mass has been studied in experimental and clinical setting, however revealing controversial results [13-17]. Evidence from experimental studies has shown that administration of thiazolidinediones might be associated with the development of LV hypertrophy [14,37,38]. Bell et al. [14] investigated the trophic effects of rosiglitazone on cardiomyocytes in an experimental in-vitro model and observed that rosiglitazone itself does not iniate cellular hypertrophy directly. However, their results might suggest that rosiglitazone in combination with growth-regulating factors may make a modest contribution to cardiac remodeling (hypertrophy). Duan et al. [38] studied the effect of rosiglitazone in cardiomyocyte-specific PPAR-γ knock-out mouse model and in control mice and observed that rosiglitazone causes cardiac hypertrophy in both models, however more pronounced in the control mice, suggesting also a partially PPAR-γ independent mechanism responsible for the hypertrophic effects. On the other hand, studies have reported that thiazolidinediones inhibit cardiac hypertrophy [13,16]. Asakawa et al. [13] evaluated the effect of thiazolidinediones in-vitro on cultured rat cardiac myocytes and in-vivo using mice (exposed to angiotensine II or pressure overload to induce hypertrophic remodeling) and observed that thiazolidinedione therapy inhibit cardiac hypertrophy.

Thus far, only few studies have evaluated the possible hypertrophic effect of thiazolidinediones in patients [15,17]. St. John Sutton et al. [17] studied patients with type 2 diabetes using echocardiography and demonstrated that long-term use of rosiglitazone is not associated with increase in LV mass (or functional impairment). Likewise, Ghazzi et al. [15] performed an echocardiographic study in patients with type 2 diabetes submitted to troglitazone therapy and could not observe an increase in LV mass either.

Accordingly, the current study is the first study that evaluated the effect of rosiglitazone therapy on LV mass-I in subjects with the metabolic syndrome using CMR and revealed that rosiglitazone inhibits the positive effects (of reverse remodeling) of intensive lifestyle intervention. Due to the observational character of this study, the underlying mechanism remains to be elucidated. Possible explanations maybe that the effect of rosiglitazone on cardiomyocytes might inhibit the process of reverse remodeling directly. Or, the concurrent hypertrophic effect of rosiglitazone may minimize the effect of reverse remodeling (reduction of LV mass) due to lifestyle intervention, resulting in unchanged LV mass. Besides a direct effect of rosiglitazone on cardiomyocytes, it is suggested that thiazolidinediones affect hemodynamics by increasing mechanical loading through different mechanisms: thiazolidinediones may increase cardiac output due to decreased afterload as a consequence of decreased peripheral resistance, and enhanced fluid retention leading to increased cardiac preload [5,14]. The effect of reverse remodeling (LV mass reduction) due to lifestyle intervention and the simultaneously occurring haemodynamic effects of rosiglitazone might cancel each other out.

In two patients from the group following rosiglitazone therapy, anti-hypertensive medication was initiated during the course of the study. Two other patients from this group were already treated with anti-hypertensive medication at baseline, as well as four patients from the placebo group. The fact that medication was initiated during the study in patients from the rosiglitazone group, would potentially imply additional LV mass reduction in this group. And since LV mass-I was not significantly different for these patients at follow-up, this observation may even add to the possible cancelling effect of rosiglitazone on reverse LV remodeling.

The fact that the previous clinical studies [15,17] were not able to find an effect of thiazolidinedione on LV mass might be explained by the use of CMR, enabling highly accurate measurements of LV mass [18] in stead of echocardiography which is operator and acoustic window dependent [39].

During the past few years, the safety of rosiglitazone therapy has been a matter of debate. Nissen and Wolski [40] performed a meta-analysis to study the effect of rosiglitazone on cardiovascular morbidity and mortality and concluded that rosiglitazone therapy was associated with an increased risk of myocardial infarction. The recently published RECORD trial evaluated the effect of addition of rosiglitazone to glucose-lowering therapy and found an increased risk of heart failure in patients treated with rosiglitazone compared to controls, whereas the overall cardiovascular morbidity and mortality was similar [41]. The results of the current study, suggesting a hypertrophic effect of rosiglitazone, and the previously recognized relation between LV hypertrophy and cardiac events such as ischemia and heart failure [12], might add to the paradigm of sodium and water retention to explain the increased risk of these events in patients using rosiglitazone.

A limitation of the present study is the relatively small study population. However, the use of CMR allows for very accurate and reproducible measurements enabling significant reduction in sample size [18].

## Conclusion

Intensive lifestyle intervention resulted in LV mass-I reduction in subjects with the metabolic syndrome, indicating reverse remodeling. However, rosiglitazone therapy may have inhibited this positive reverse remodeling (LV mass-I reduction).

## Competing interests

The authors declare that they have no competing interests.

## Authors' contributions

SDR participated in the study design, carried out the data collection and analysis and drafted the manuscript; RAD participated in the study design and data collection; JJMW participated in the study design, data collection and analysis; HJL participated in the study design; BJAM participated in the study design and carried out the statistical analysis, JTT participated in the study design, AdR participated in the study design and interpretation of the results. All authors read and approved the final manuscript.
